# Increasing the Availability and Consumption of Drinking Water in Middle Schools: A Pilot Study

**Published:** 2011-04-15

**Authors:** Anisha I. Patel, Laura M. Bogart, David J. Klein, Mark A. Schuster, Marc N. Elliott, Jennifer Hawes-Dawson, Sheila Lamb, Kimberly E. Uyeda

**Affiliations:** Assistant Professor, Department of Pediatrics, University of California at San Francisco. Dr Patel is also affiliated with the Philip R. Lee Institute for Health Policy Studies, San Francisco, California; Division of General Pediatrics, Children's Hospital Boston, Boston, Massachusetts, Harvard Medical School, Boston, Massachusetts, and RAND Corporation, Santa Monica, California; Division of General Pediatrics, Children's Hospital Boston, Boston, Massachusetts, Harvard Medical School, Boston, Massachusetts, and RAND Corporation, Santa Monica, California; Division of General Pediatrics, Children's Hospital Boston, Boston, Massachusetts, Harvard Medical School, Boston, Massachusetts, and RAND Corporation, Santa Monica, California; RAND Corporation, Santa Monica, California; RAND Corporation, Santa Monica, California; Los Angeles Unified School District, Los Angeles, California; Los Angeles Unified School District, Los Angeles, California

## Abstract

**Introduction:**

Although several studies suggest that drinking water may help prevent obesity, no US studies have examined the effect of school drinking water provision and promotion on student beverage intake. We assessed the acceptability, feasibility, and outcomes of a school-based intervention to improve drinking water consumption among adolescents.

**Methods:**

The 5-week program, conducted in a Los Angeles middle school in 2008, consisted of providing cold, filtered drinking water in cafeterias; distributing reusable water bottles to students and staff; conducting school promotional activities; and providing education. Self-reported consumption of water, nondiet soda, sports drinks, and 100% fruit juice was assessed by conducting surveys among students (n = 876), preintervention and at 1 week and 2 months postintervention, from the intervention school and the comparison school. Daily water (in gallons) distributed in the cafeteria during the intervention was recorded.

**Results:**

After adjusting for sociodemographic characteristics and baseline intake of water at school, the odds of drinking water at school were higher for students at the intervention school than students at the comparison school. Students from the intervention school had higher adjusted odds of drinking water from fountains and from reusable water bottles at school than students from the comparison school. Intervention effects for other beverages were not significant.

**Conclusion:**

Provision of filtered, chilled drinking water in school cafeterias coupled with promotion and education is associated with increased consumption of drinking water at school. A randomized controlled trial is necessary to assess the intervention's influence on students' consumption of water and sugar-sweetened beverages, as well as obesity-related outcomes.

## Introduction

Childhood obesity has increased over the past 4 decades (1). A growing literature links sugar-sweetened beverage (SSB) and 100% fruit juice consumption to obesity (2,3), and several studies suggest that drinking water helps to prevent obesity (4-6).

Because school is a primary location, ranking second only to the home, at which children consume SSBs (7), attention has focused on restricting SSB availability in schools (8). Efforts to increase access to healthy beverages, such as increasing school drinking water availability, have received less attention. Although a few European intervention studies have examined the effect of school drinking water provision and promotion on SSB consumption and childhood obesity (5,9), these findings may not be generalizable to US schools (eg, some European schools do not serve lunch or sell beverages).

In the Los Angeles Unified School District (LAUSD), the second largest US school district, water is typically available at no cost through school drinking fountains, and bottled water is sold through school vending in most middle and high schools. In 2002, the LAUSD school board passed the Motion to Promote Healthy Beverage Sales (10). Since then, beverages with less added sugar have been made available in schools. Plain and flavored nonfat or 1% milk and 100% fruit juice are provided through the National School Lunch Program (NSLP), a federal program that daily provides reduced-price and free meals to students (11). Sports drinks, 100% fruit and vegetable juices, and plain and flavored milk are sold in school stores and vending machines.

In previous studies we have conducted as a part of community-based participatory research (CBPR) to address disparities in obesity among middle school students, we observed few students drinking water from school fountains. We also found that school staff, health and nutrition agency representatives, and families voiced concerns about school water, including the appeal, taste, appearance, and safety of fountain water and the affordability and environmental effect of bottled water sold in schools (12-15). These same people also expressed interest in improving the provision of safe, palatable drinking water in schools.

Although some US schools have established programs to encourage student water consumption (12), we are aware of no published evaluation of such programs. We examined whether provision of drinking water, coupled with education and promotional activities, was related to increased consumption of water and decreased consumption of SSBs among middle school students in Los Angeles, California. A second aim was to develop a feasible and sustainable program to encourage student consumption of drinking water.

## Methods

### Design and participants

The quasi-experimental study took place in the spring of 2008 and tested a 5-week pilot intervention to increase drinking water availability and consumption among students in 1 LAUSD middle school. In selecting a school for the pilot test, we considered only schools in which at least 60% of students qualified for free and reduced-price NSLP meals (a proxy for household income) because of the high prevalence of obesity among adolescents of low socioeconomic status (1). We also considered school interest and a preexisting relationship with the research team. We selected the comparison school on the basis of its comparability to the intervention school (Table 1); we chose 1 from among 4 schools that were in the same geographic area and had similar numbers of students, student racial/ethnic composition, and percentage of students who were learning English.

Although the intervention included schoolwide activities that could affect all students' beverage intake, because of cost limitations, we surveyed only 7th-grade students at the intervention and comparison schools.

We recruited study participants through 7th-grade science classes. Research staff distributed study information and consent forms written in English and Spanish for parent or guardian signature and returned to schools 3 times to redistribute information and collect completed materials. Students in classes that returned at least 80% of consent forms (signed yes or no) received $5 gift cards. Although 7th-grade enrollment status, proficiency in English, and parental consent were required for survey participation, all 6th- through 8th-grade students at the intervention school were eligible for intervention activities. The RAND and LAUSD institutional review boards approved the study.

### Intervention components

This study took place within the context of a larger CBPR study to address disparities in adolescent obesity (12-15). Formative research (12-14), recommendations from intervention school personnel and students, and input from community advisory boards (15) informed intervention development. Intervention components were the provision of cold, filtered tap water in the school cafeteria; distribution of reusable water bottles to all school staff and students; implementation of school-wide promotional activities; and education regarding the benefits of drinking water.

**Drinking water provision**

For the intervention, cafeteria staff filled 5-gallon dispensers (Figure) with filtered tap water from a cafeteria faucet. In accordance with Environmental Protection Agency (EPA) guidelines, we sent a 250-mL water sample obtained from the faucet after a 6- to 8-hour period of nonuse to an EPA-certified laboratory for testing (17). The lead level for the tested water sample was less than the EPA action level (15 ppb). A water treatment company installed a carbon coconut shell and 5-micrometer sediment filter on a cafeteria faucet to improve the taste and appearance of the dispensed tap water.

**Figure:**
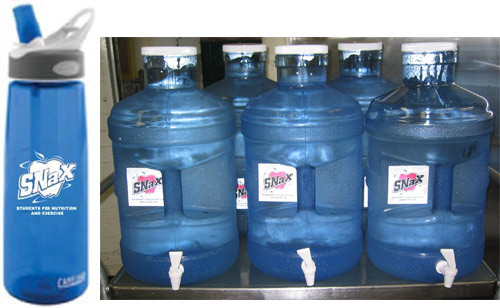
Water bottle and filtered tap water dispensed as part of school environmental changes to promote student water intake, Los Angeles, California, 2008.

Cafeteria staff filled dispensers with filtered tap water, refrigerated them, and placed them in the cafeteria courtyard during mealtimes. Cafeteria staff sanitized water dispensers weekly according to a protocol developed by cafeteria staff and research team members. At the start of the intervention, all students and staff received a reusable water bottle with a school and study logo (Figure) to fill from the 5-gallon dispensers. Teachers instructed students to write their name on their bottles, fill the bottles only with water, and regularly wash the bottles.

We monitored student cafeteria water consumption at the intervention school throughout the 5-week intervention period and tested alternative strategies to increase water consumption. During intervention week 4, we placed paper cups next to water dispensers for students who did not have their water bottles. We also visited the intervention school after the intervention to observe the extent to which cafeteria staff continued to offer students water.

**Promotional activities**

We held school promotional activities to encourage student water consumption. We entered students and staff seen drinking water from cafeteria dispensers in a weekly drawing for prizes. Students made public address announcements to promote intervention activities and encourage water consumption. The school held art contests to engage students in developing messages regarding the healthfulness of drinking tap water rather than SSBs.

**Educational activities**

Educational activities included posting nutritional information for beverages available in the school cafeteria or store; posting and distributing posters, bookmarks, and flyers with messages about the health and environmental benefits of drinking tap water instead of bottled water or SSBs (eg, if you drink free water instead of buying a drink every day, in 6 months you would have saved enough money to buy an iPod); conducting educational sessions about the benefits of drinking tap water instead of SSBs (1 session for approximately 30 parents and 1 session for all school employees); and conducting an educational session for 3 interested 7th-grade science classes about drinking water quality, which included hands-on lead testing of water from selected school drinking fountains.

### Measures

**Student surveys**

Questions from previously validated surveys were used for the study questionnaire (18,19). When validated measures did not exist, we developed new questions from qualitative research on drinking water provision in schools (12-14). We refined survey questions based on hour-long cognitive interviews in which 6th and 8th graders from the intervention school read survey questions aloud, reflected on the meaning of questions, and highlighted difficulties in question comprehension (20).

To assess school water intake, students were asked whether they drank water at school from each of the following sources the day before the survey: 1) a fountain, 2) a sink or faucet, 3) a bottle, 4) a reusable water bottle brought from home, or 5) another source. Students also specified whether they drank any of the following the day before the survey: 1) nondiet sodas, 2) sports drinks, or 3) 100% fruit juice.

Students in intervention and comparison schools completed self-administered surveys during science classes preintervention and at 1 week and 2 months after the 5-week intervention. We held make-up sessions at each school 1 week after the regularly scheduled surveys to capture students who were absent during the initial survey administration. Follow-up surveys at the intervention school assessed intervention feasibility and sustainability. This survey asked students why they do not bring the reusable water bottle to school (eg, I forget to bring it; it is too big or heavy), about drinks they put in reusable water bottles (eg, water from the cafeteria or drinking fountains at school, regular soda [nondiet]), and to rate various intervention components on a scale from 1 to 5 (1 being poor and 5 being excellent).

All surveys assessed sociodemographic characteristics of the students (ie, race/ethnicity, age, primary language spoken at home, sex, and eligibility for free and reduced-price lunch through the NSLP).

**Cafeteria records**

Cafeteria staff recorded the daily amount of water taken from dispensers at mealtimes during the 5-week intervention. Cafeteria staff also documented daily the staffing time required to provide drinking water (ie, to fill, sanitize, and transport dispensers).

### Statistical analyses

We calculated means and standard errors and used 2-sample *t *tests to compare outcome variables by intervention and comparison school. We used multivariate logistic regression models to predict the odds of drinking water, nondiet soda, sports drinks, and 100% fruit juice at school on the previous day and at 1 week and 2 months postintervention, separately, controlling for intervention status, preintervention student consumption of drinking water at school, age, sex, race/ethnicity, primary language spoken at home, and NSLP eligibility. We used descriptive statistics to determine mean amount of water (in gallons) taken from 5-gallon dispensers in the cafeteria and student ratings of and reasons for not bringing reusable water bottles to school.

We used Stata version 10 (StataCorp LP, College Station, Texas) to perform multivariate analyses and SAS version 9.1.3 (SAS Institute, Inc, Cary, North Carolina) to impute missing student survey data (21). We used student responses to all items from all survey waves (ie, preintervention, 1 week postintervention, and 2 months postintervention) to impute missing data.

## Results

### Study participants

Although student surveys were conducted at 1 week and 2 months postintervention, only the 2-month postintervention results are reported here because they are most indicative of intervention sustainability. Written parental consent was received for 77% (n = 419) of students from the intervention school and for 79% (n = 484) of students from the comparison school. A total of 7% of parents (6% from the intervention school, 7% from the comparison school) actively declined on the consent forms to allow their children to participate. Of students with parental consent, 97% from both schools participated in the preintervention assessment. Postintervention (2 months) surveys were completed by 90% (n = 793) of preintervention participants (90% from the intervention school, 91% from the comparison school). Of the 83 students who completed preintervention assessments but did not complete the 2-month postintervention survey, 42% were absent, 34% transferred to another school, 17% declined, and 7% otherwise did not complete the survey.

Intervention and comparison schools did not significantly differ with regard to student age, sex, or NSLP eligibility, but there were differences in racial/ethnic distribution and in language spoken at home (Table 2). Compared with 6th- through 8th-grade students overall (Table 1), a higher percentage of 7th-grade students reported being of other race/ethnicity; a lower percentage reported being eligible for free and reduced-price meals through the NSLP.

### Outcomes

**Student beverage consumption**

At 2 months postintervention, the unadjusted change between students in the comparison school and students at the intervention school who reported drinking any water at school was 9 percentage points (−3.7 to 5.7) (Table 3). The relative change in any water consumption between the 2 schools remained significantly different after adjustment (*P* = .003). Regarding student water consumption from different sources at school, the unadjusted change between students at the intervention school and students at the comparison school who reported drinking water from school drinking fountains was approximately 9 percentage points (−2.6 to 6.0), and the unadjusted change between students at the intervention school and students at the comparison school who reported drinking water from reusable water bottles was approximately 8 percentage points (−1.7 to 6.1). The relative change remained significant for water consumption from drinking fountains (*P* = .02) and reusable water bottles (*P* = .005) after adjustment. No other significant differences between intervention and comparison schools were found.

**Water distributed from cafeteria dispensers**

During the first week of the intervention (when students and staff received reusable water bottles), the mean amount of water taken from cafeteria water dispensers was 31 gallons per day or 0.3 cup per student per day. This amount decreased substantially by week 5 to 10 gallons per day or 0.1 cup per student per day.

**Intervention sustainability**

At 1 week and 2 months postintervention, only 13% and 9% of students surveyed, respectively, reported bringing their reusable water bottle to school to drink water. The most commonly reported reasons for not bringing them were that students forgot to bring them (41%), the bottles were too heavy to carry (36%), the bottles were not "cool" (30%), and students preferred commercial bottled water to tap water (29%). Most students rated the drinking water from the cafeteria dispensers (88%) and the reusable water bottles distributed during the intervention (86%) as good, very good, or excellent. Water was the most commonly reported beverage that students consumed from reusable water bottles (63%). Other beverages were 100% fruit juice (24%), sports drinks (23%), and nondiet soda (21%); 39% of intervention school students reported using their reusable water bottle to drink at least 1 SSB in the last month.

Although the study ended in March 2008, cafeteria staff continued to provide filtered, chilled tap water to students at lunch after the intervention (March 2008-December 2009). Staff also used cafeteria funds to provide free cups during warm weather so that students without water bottles could get water from the dispensers at lunch. In September 2010, due in part to advocacy efforts by our community partners, Governor Arnold Schwarzenegger signed Senate Bill 1413, legislation that will require California school districts to offer fresh, free drinking water in food service areas of California public schools by July 2011 (22).

## Discussion

This pilot study suggests that provision of cold, filtered drinking water in 5-gallon dispensers in school cafeterias coupled with promotional and educational activities can increase water consumption among middle-school students. Intervention school students had significantly higher odds of drinking water from school drinking fountains and from reusable water bottles at school than did comparison school students. Although water was distributed from cafeteria water dispensers for the duration of the 5-week program, the amount of water dispensed decreased over the length of the program as student use of reusable water bottles declined (at 2 months postintervention, less than 10% of intervention school students reported using them).

Previous European intervention studies associated school drinking water provision and promotion with an increase in student water consumption but no change in student SSB consumption or school soft drink sales (5,9). One of the studies demonstrated a decreased risk of overweight (defined by continuous body mass index [BMI] standard deviation scores or deviation from the median of the sex- and age-independent BMI distribution) among students from the intervention school relative to students from the control group (5).

Although reusable water bottles were an effective means for encouraging water consumption in a German elementary school study in which students were allowed to store reusable water bottles at school (5), in both our study and an English secondary school study (9), reusable water bottles did not prove to be such an effective strategy. Perhaps reusable water bottles may be a more appropriate strategy for encouraging water consumption among students in schools that provide storage for bottles so that they are less likely to be lost, damaged, or forgotten at home. For schools that lack such storage, providing water coolers with paper cups or free bottled water with meals may be more effective tactics to encourage water consumption. In our study, research staff observed that when paper cups were placed next to water dispensers during the intervention, the amount of water taken from 5-gallon dispensers in the cafeteria increased.

Although our study included education and promotional activities to encourage student water consumption, the duration of such events was limited to 5 weeks. Whereas a previous study engaged teachers to help students fill up their water bottles each morning at school (5), here teachers and parents were not as fully active in the intervention.

An investigative report that publicized elevated lead levels found in tap water at some LAUSD schools that was made public during the intervention also may have decreased the intervention's effectiveness. Data from the comparison school demonstrated an unexpected decrease in water consumption at school from pre- to postintervention, during a period that coincides with the investigative report.

Although we hypothesized that this pilot study would decrease intervention students' consumption of SSBs, we did not observe such an effect. This may have been secondary to low baseline student consumption of SSBs due to preexisting LAUSD policies that have limited the availability of SSBs on school campuses. Alternatively, some students' use of reusable water bottles to drink SSBs may have limited the intervention's effectiveness.

The ultimate aim of an intervention that encourages drinking water provision in schools is to affect clinical outcomes such as BMI. Because this was a small quasi-experimental pilot study in which a causal relationship between the intervention and obesity could not be determined, we did not measure participants' BMI. Our goal was to develop a strategy for encouraging drinking water consumption in a large urban US school district. Another limitation of this study was the use of self-reported student data to measure beverage consumption. Future studies should consider using additional means to evaluate beverage intake, such as observation of students or the use of flow meters to determine the amount of drinking water dispensed from drinking water outlets.

Results from this study suggest that provision of filtered, chilled drinking water in school cafeterias coupled with promotion and education efforts may be an effective means for increasing student consumption of drinking water in school. Future studies are needed to explore the most effective and cost-effective ways to encourage drinking water consumption among students from different age groups and in different settings. Although empirical support is emerging that drinking water provision in schools may prevent overweight, future studies are necessary to investigate how schools can best implement programs and which components (education, promotion, environmental change) are most effective in improving student consumption of drinking water.

## Figures and Tables

**Table 1:** Sociodemographic Characteristics of Intervention and Comparison Middle Schools, Los Angeles, California, 2008^a^

**Characteristic**	Intervention School (n = 1,669), %	Comparison School (n = 1,924), %
**Race/ethnicity**		
API/other	23	23
African American	19	9
Hispanic	53	62
**English learners^b^**	15	18
**Eligible for NSLP^c^**	72	66

Abbreviations: API, Asian or Pacific Islander; NSLP, National School Lunch Program.

a Data obtained from Education Data Partnership (16).

b Students who report a primary language other than English and who have been determined by the state of California to lack clearly defined English language skills necessary to succeed in the school's regular instructional programs.

c Refers to students who are eligible for free or reduced-cost lunch through the NSLP.

**Table 2:** Baseline Characteristics of Study Participants From Intervention and Comparison Middle Schools in Los Angeles, California, 2008

**Variable**	Intervention School (n = 405)	Comparison School (n = 471)	*P* Value^a^
**Mean age, y (SD)**	12.8 (0.75)	12.9 (0.46)	.27
**Female, %**	56	54	.57
**Race/ethnicity, %**			
Hispanic	53	63	.001
Asian/Pacific Islander	22	22	.90
African American	14	6	.001
Other	11	9	.31
**Language(s) spoken at home, %**			
English only	37	29	.01
English plus another	50	55	.11
No English	10	14	.05
**NSLP eligibility,^b^ %**	63	63	.92

Abbreviation: NSLP, National School Lunch Program.

a *P* values are based on 2-sample *t-*tests that compared sociodemographic variables by school (intervention vs comparison).

b Refers to students who are eligible for free or reduced-cost lunch through the NSLP.

**Table 3:** Consumption of Water, Nondiet Soda, Sports Drinks, and 100% Fruit Juice Among Los Angeles Middle School Students, Preintervention and 2 Months Postintervention, 2008

Behavior on the Previous Day	Preintervention, n (%)^a^	2 Months Postintervention, n (%)^a^	Percentage Change, Unadjusted	*P* Value^b^	AOR (95% CI)	*P* Value^c^
**Drank any water at school**						
Comparison	340 (79.1)	324 (75.4)	−3.7	.006	1.76 (1.20-2.57)	.003
Intervention	279 (76.9)	300 (82.6)	5.7			
**Drank water from school fountain **						
Comparison	235 (54.7)	224 (52.1)	−2.6	.03	1.45 (1.05-1.99)	.02
Intervention	185 (51.0)	207 (57.0)	6.0			
**Drank water from other tap water source**						
Comparison	16 (3.7)	30 (7.0)	3.3	.14	1.59 (0.93-2.73)	.09
Intervention	16 (4.4)	39 (10.7)	6.3			
**Drank bottled water**						
Comparison	133 (30.9)	142 (33.0)	2.1	.65	1.03 (0.75-1.41)	.87
Intervention	125 (34.4)	126 (34.7)	0.3			
**Drank water from reusable water bottle**						
Comparison	45 (10.5)	38 (8.8)	−1.7	.003	1.99 (1.23-3.20)	.005
Intervention	35 (9.6)	57 (15.7)	6.1			
**Drank any soda**						
Comparison	219 (50.9)	241 (56.1)	5.2	.10	0.89 (0.66-1.20)	.46
Intervention	202 (55.7)	195 (53.7)	−2.0			
**Drank any sports drink **						
Comparison	229 (53.3)	216 (50.2)	−3.1	.12	1.31 (0.97-1.75)	.08
Intervention	185 (51.0)	199 (54.8)	3.8			
**Drank any 100% fruit juice **						
Comparison	185 (43.0)	136 (31.6)	−11.4	.01	1.28 (0.94-1.76)	.12
Intervention	129 (35.5)	127 (35.0)	−0.5			

Abbreviations: AOR, adjusted odds ratio; CI, confidence interval.

a Values are unadjusted percentages.

b *P* values were calculated by using paired *t* tests for differences in change from preintervention to postintervention (between intervention school and comparison school).

c *P* values were calculated by using multivariable logistic regression models to predict the odds of drinking various beverages at 2 months postintervention, separately, controlling for intervention status, preintervention student consumption of beverages at school, age, sex, race/ethnicity, primary language spoken at home, and National School Lunch Program eligibility.
